# Successful pembrolizumab treatment of microsatellite instability‐high intrahepatic cholangiocarcinoma: A case report

**DOI:** 10.1002/ccr3.4008

**Published:** 2021-03-04

**Authors:** Yuki Ikeda, Michihiro Ono, Ginji Ohmori, Saki Ameda, Michiko Yamada, Tomoyuki Abe, Shigeyuki Fujii, Miri Fujita, Masahiro Maeda

**Affiliations:** ^1^ Department of Gastroenterology Steel Memorial Muroran Hospital Muroran Japan; ^2^ Department of Pathology and Clinical Laboratory Steel Memorial Muroran Hospital Muroran Japan

**Keywords:** cholangiocarcinoma, microsatellite instability‐high, pembrolizumab

## Abstract

The tumor reduction effect of pembrolizumab is extremely high compared to standard chemotherapy and might show prolonged survival. Therefore, the MSI status should be examined in patients with cholangiocarcinoma.

## Graphical abstract

Contrast‐enhanced computed tomography (CT) revealed a primary tumor (black arrow) before the initiation of pembrolizumab (A). After three cycles of pembrolizumab, the primary tumor showed a 32% reduction (B). After 14 cycles, the primary tumor shoed a 79% reduction (C).

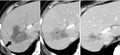

## 1 INTRODUCTION

Pembrolizumab is considered to be an effective therapy for patients with microsatellite instability (MSI)‐high cancer. Here, we report a case of MSI‐high cholangiocarcinoma effectively treated with pembrolizumab. MSI status should be actively evaluated in patients with cholangiocarcinoma.

Cholangiocarcinoma is considered to be intractable and has a poor prognosis. Gemcitabine plus cisplatin is recommended as first‐line chemotherapy for patients with advanced cholangiocarcinoma based on an ABC‐02 study.^1^ The median overall survival (OS) after gemcitabine and cisplatin treatment is 11.7 months compared to gemcitabine monotherapy, which is 8.1 months. Furthermore, the median OS of gemcitabine/cisplatin/S‐1 (GCS) combination therapy was 16.2 months, compared to gemcitabine and cisplatin therapy, and is becoming one of the standard chemotherapy for advanced cholangiocarcinoma in Japan.^2^

Recently, immune checkpoint inhibitors (ICIs) have shown promising responses in cancer treatment, regardless of primary site.^3^ Pembrolizumab, a monoclonal antibody against programmed cell death protein (PD‐1), was approved for patients with microsatellite instability (MSI)‐high and mismatch repair‐deficient (dMMR) cancer. However, only a few cases have had pembrolizumab administered for advanced cholangiocarcinoma.

Here, we present a case of MSI‐high intrahepatic cholangiocarcinoma (ICC) in which the tumor was markedly reduced by pembrolizumab treatment.

## 2 CASE REPORT

**FIGURE 1:**
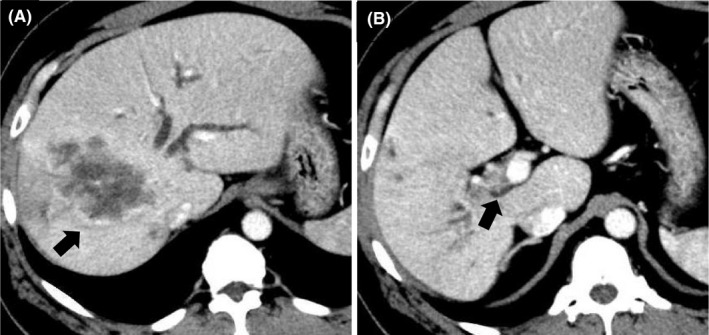
Contrast‐enhanced computed tomography (CT) revealed a 98‐mm tumor (black arrow) in segment 7/8 of the liver (A) and also a right portal vein tumor thrombosis (black arrow) (B)

**FIGURE 2:**
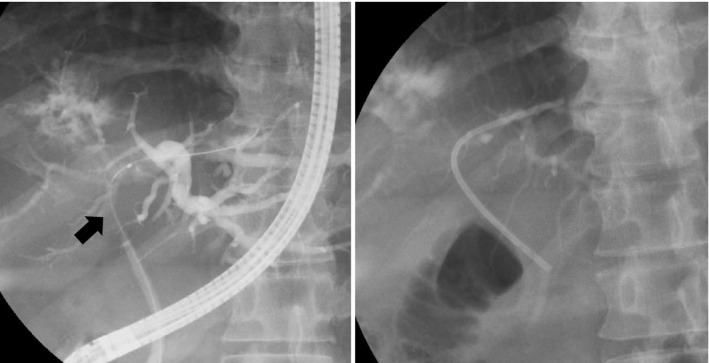
Endoscopic retrograde cholangiopancreatography revealed a stricture in the hilar bile duct (black arrow) (A). A plastic stent was placed through the biliary stricture (B)

**FIGURE 3:**
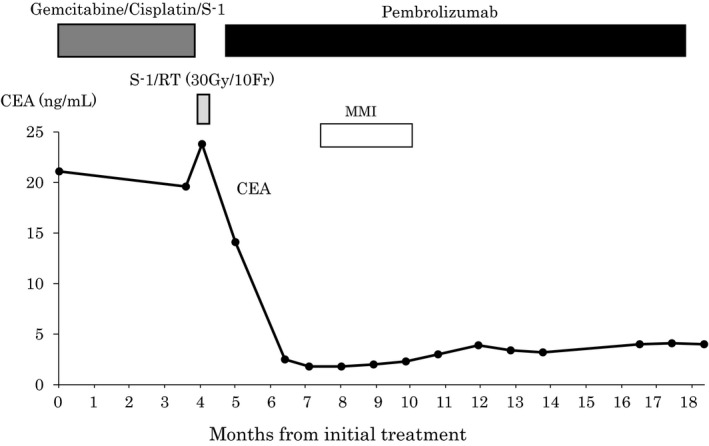
Clinical course. This graph demonstrates changes in the carcinoembryonic antigen level (CEA) level and the timeline from initial treatment

**FIGURE 4:**
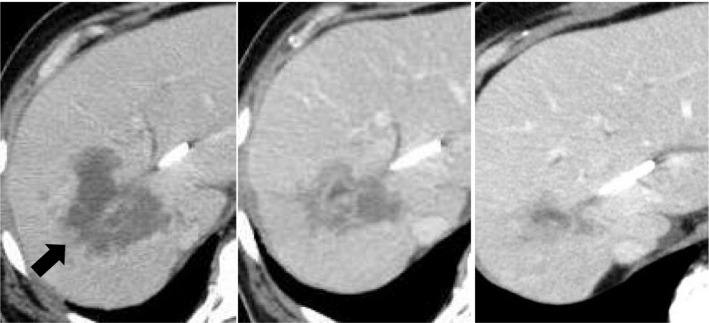
Contrast‐enhanced computed tomography (CT) revealed a primary tumor (black arrow) before the initiation of pembrolizumab (A). After three cycles of pembrolizumab, the primary tumor showed a 32% reduction (B). After 14 cycles, the primary tumor showed a 79% reduction (C)

A 50‐year‐old man presented with epigastric and back pain and was admitted to hospital. Laboratory data revealed alkaline phosphatase (ALP) 824 U/L (106‐322 U/L), ɤ‐GTP 401 U/L (5‐50 U/L) and carcinoembryonic antigen (CEA) 21.1 ng/mL (0‐5.0 ng/mL; Table 1). Abdominal computed tomography (CT) revealed a 98‐mm tumor in segment 7/8 of the liver with right portal vein tumor thrombosis (PVTT) and dilation of the left intrahepatic bile duct (Figure 1A, 1B). Endoscopic retrograde cholangiopancreatography showed a stricture in the hilar bile duct (Figure 2A). A plastic stent (7 Fr, 12 cm at a deep angle) was placed in the biliary stricture (Figure 2B). Biopsy specimens revealed an adenocarcinoma. However, the patient was diagnosed with an unresectable tumor due to insufficient residual liver volume and was administered GCS combination therapy as outlined in Figure 3. After six cycles, the CEA level was found to be elevated at 23.8 ng/mL, with CT showing an enlarged right PVTT. Oral S‐1 combined with radiotherapy (36 Gy/12 fractions) was initiated as second‐line therapy for the right PVTT; however, the liver tumor was enlarged to 102 mm. (Figure 4A).

**TABLE 1:** Laboratory data

<Peripheral blood>		<Blood chemistry>		<Tumor markers>	
WBC	8160/μL	TP	6.4 g/dL	CEA	21.1 ng/mL
RBC	370 × 10^4^/μL	Alb	3.0 g/dL	CA19‐9	2.5 U/mL
Hb	11.7 g/dL	T. Bil	0.9 mg/dL		
Ht	35.4%	D. Bil	0.3 mg/dL		
Plt	28.2 × 10^4^/μL	AST	33 U/L		
		ALT	29 U/L		
<Coagulation>		LDH	194 U/L		
PT‐INR	0.95 s	ALP	824 U/L		
APTT	27.3 mg/dL	γ‐GTP	401 U/L		
FBG	384	AMY	50 U/L		
		BUN	8.8 mg/dL		
		Cr	0.71 mg/dL		
		Na	142 mEq/L		
		K	3.8 mEq/L		
		Cl	107 mEq/L		
		Ca	8.8 mg/dL		
		CRP	4.76 mg/dL		
		FBS	131 mg/dL		
		HbA1c	5.0%		

MSI status was investigated using an approved kit (MSI‐IVD kit, FALCO biosystems). An analysis of liver tumor biopsy specimens showed a high MSI status; the patient was administered pembrolizumab (200 mg, every 3 weeks). After three cycles of pembrolizumab, the CEA level declined to within normal range (2.3 ng/mL), with CT showing a reduction of 69mm and 32% primary tumor reduction (Figure 4B), leading to a partial response (PR). After 14 cycles, CT revealed a reduction of 21mm and 79% primary tumor reduction (Figure 4C), maintaining the PR; however, the PVTT remained. Four cycles after initiating pembrolizumab, grade 2 hyperthyroidism was observed as an immune‐related adverse event. Methimazole was administered for three months, and the patient's thyroid function improved.

## 3 DISCUSSION

We report a case of advanced cholangiocarcinoma treated with ICI that showed a high response to pembrolizumab monotherapy after standard chemotherapy. Pembrolizumab, is an anti–PD‐1 monoclonal antibody that has shown antitumor activity in various types of cancers, including nonsmall‐cell lung cancer, gastric cancer, urothelial cancer, and melanoma.^4^, ^5^, ^6^, ^7^ MSI‐high/dMMR is considered a potential biomarker of the response to pembrolizumab in several types of cancers.^8^ Pembrolizumab was approved for previously treated unresectable or metastatic MSI‐high/dMMR cancers in Japan. Based on a phase II KEYNOTE‐158 study that enrolled 233 patients with MSI‐high/dMMR noncolorectal cancer, the objective response rate (ORR) was 34.3% (95% confidence interval [CI], 28.3‐40.8%), the median progression‐free survival (PFS) was 2.1 months (95% CI, 2.4 to 4.9 months), and OS was 23.5 months (95% CI, 13.5 months to not reached) for MSI‐high/dMMR noncolorectal cancer.^3^

Only three case reports exist of MSI‐high advanced cholangiocarcinoma treated with pembrolizumab.^9^, ^10^, ^11^ All cases showed a good response and maintained a PR during pembrolizumab treatment. In prior studies of pembrolizumab treatment for advanced cholangiocarcinoma,^3^ pembrolizumab showed a certain efficacy, with a ORR of 6‐17%, median PFS of 1.5‐2.1 months, and a median OS of 4.3‐7.4 months for a negative or unknown MSI status^12^, ^13^, ^14^, ^15^ (Table 2). However, the KEYNOTE‐158 study,^3^ which included 22 patients with MSI‐high cholangiocarcinoma, showed a better ORR of 40.9% (95% CI, 20.7‐63.6%), and longer median PFS and OS of 4.2 months (95% CI, 2.1 months to not reached) and 24.3 months (95% CI, 4.1 to 24.9 months), respectively, compared to patients with MSI‐negative or unknown. Pembrolizumab in patients with MSI‐high cholangiocarcinoma showed a better therapeutic effect than in patients with an unknown MSI status. Compared with gemcitabine plus cisplatin in the ABC‐02 study,^1^ pembrolizumab induced better tumor shrinkage and prolonged survival (24.3 months vs 11.7 months). Thus, pembrolizumab should be considered for first‐line therapy for MSI‐high advanced cholangiocarcinoma in future.

**TABLE 2:** Clinical trials of pembrolizumab monotherapy for cholangiocarcinoma

References	N	MSI, %	ORR(%)	PFS(M)	OS(M)
Bang et al^13^	24	NA	17	NA	NA
Lee et al^14^	51	NA	10	2.1	6.9
Kang et al^15^	40	NA	10	1.5	4.3
Piha‐Paul et al^16^	104	Negative, 95	6	2	7.4
		Missing, 5			
Marabelle et al^4^	22	High, 100	41	4.2	24.3

Note

Abbreviations: NA, not available; ORR, overall response rate; OS, overall survival; PFS, progression‐free survival.

In our case, pembrolizumab monotherapy for ICC refractory to GCS combination therapy was found to be effective, with a PR maintained 13 months after treatment. The frequency of MSI‐high in all cholangiocarcinomas is only 2%,^16^ and the rate for benefiting from pembrolizumab is low. However, since patients with MSI‐high cholangiocarcinoma administered pembrolizumab might show prolonged survival compared to standard chemotherapy, it is necessary to examine the MSI status in order to identify patients that might benefit from pembrolizumab treatment.

## 4 CONCLUSION

MSI‐high cases are rare among patients with advanced cholangiocarcinoma; however, the potential therapeutic effect of pembrolizumab is extremely high. Therefore, the MSI status of advanced cholangiocarcinoma cases should be actively examined since some patients can benefit from pembrolizumab treatment.

## AUTHOR CONTRIBUTIONS

YI and MO: collected data and wrote and edited the manuscript. GO, SA, MY, TA, and SF: involved in the patient's care. MF: Pathologist who diagnosed this disease. MM: supervised this study.

## CONFLICT OF INTEREST

The authors declare that they have no conflicts of interest.

## AUTHOR CONTRIBUTIONS

YI and MO: collected data and wrote and edited the manuscript. GO, SA, MY, TA, and SF: involved in the patient's care. MF: Pathologist who diagnosed this disease. MM: supervised this study.

## Data Availability

Data available within the article.

## References

[1] Valle J , Wasan H , Palmer DH , et al. Cisplatin plus gemcitabine versus gemcitabine for biliary tract cancer. N Engl J Med. 2010;362:1273‐1281. 20375404 10.1056/NEJMoa0908721

[2] Kanai M , Hatano E , Kobayashi S , et al. A multi‐institution phase II study of gemcitabinecisplatin/S‐1 (GCS) combination chemotherapy for patients with advanced biliary tract cancer (KHBO 1002). Cancer Chemother Pharmacol. 2015;75:293‐300. 25477010 10.1007/s00280-014-2648-9

[3] Marabelle A , Le DT , Ascierto PA , et al. Efficacy of pembrolizumab in patients with noncolorectal high microsatellite instability/mismatch repair‐deficient cancer: results from the phase II KEYNOTE‐158 study. J Clin Oncol. 2019;38:1‐10. 31682550 10.1200/JCO.19.02105 PMC8184060

[4] Garon EB , Rizvi NA , Hui R , et al. Pembrolizumab for the treatment of non‐small‐cell lung cancer. N Engl J Med. 2015;372:2018‐2028. 25891174 10.1056/NEJMoa1501824

[5] Muro K , Chung HC , Shankaran V , et al. Pembrolizumab for patients with PD‐L1‐positive advanced gastric cancer (KEYNOTE‐012): a multicentre, open‐label, phase 1b trial. Lancet Oncol. 2016;17:717‐726. 27157491 10.1016/S1470-2045(16)00175-3

[6] Bellmunt J , de Wit R , Vaughn DJ , et al. Pembrolizumab as second‐line therapy for advanced urothelial carcinoma. N Engl J Med. 2017;376:1015‐1026. 28212060 10.1056/NEJMoa1613683 PMC5635424

[7] Eggermont AM , Blank CU , Mandala M , et al. Adjuvant pembrolizumab versus placebo in resected stage III melanoma. N Engl J Med. 2018;378:1789‐1801. 29658430 10.1056/NEJMoa1802357

[8] Yarchoan M , Hopkins A , Jaffee EM . Tumor Mutational Burden and Response Rate to PD‐1 Inhibition. N Engl J Med. 2017;377:2500‐2501. 29262275 10.1056/NEJMc1713444 PMC6549688

[9] Czink E , Kloor M , Goeppert B , et al. Successful immune checkpoint blockade in patient with advanced stage microsatellite‐unstable biliary tract cancer. Cold Spring Harb Mol Case Stud. 2017;3:a001974. 28619747 10.1101/mcs.a001974 PMC5593153

[10] Nakamura M , Ueno M , Hayami S , et al. Effective response of intrahepatic cholangiocarcinoma to pembrolizumab: a case report. Anticancer Res. 2020;40:4123‐4129. 32620661 10.21873/anticanres.14411

[11] Naganuma A , Sakuda T , Murakami T , et al. Microsatellite Instability‐high Intrahepatic Cholangiocarcinoma with Portal Vein Tumor Thrombosis Successfully Treated with Pembrolizumab. Intern Med. 2020;18:2261‐2267. 10.2169/internalmedicine.4588-20 PMC7578609 32536644

[12] Bang YJ , Doi T , Braud FD , et al. 525 Safety and efficacy of pembrolizumab (MK 3475) in patients with advanced biliary tract cancer: Interim results of KEYNOTE‐028. Eur J Cancer. 2015;51:S112.

[13] Lee SH , Lee HS , Lee SH , et al. Efficacy and safety of pembrolizumab for gemcitabine/cisplatin‐refractory biliary tract cancer: a multicenter retrospective study. J Clin Med. 2020;9:1769. 10.3390/jcm9061769 PMC7355970 32517311

[14] Kang J , Jeong JH , Hwang HS , et al. Efficacy and safety of pembrolizumab in patients with refractory advanced biliary tract cancer: tumor proportion score as a potential biomarker for response. Cancer Res Treat. 2020;52:594‐603. 32019287 10.4143/crt.2019.493 PMC7176957

[15] Piha‐Paul SA , Oh DY , Ueno M , et al. Efficacy and safety of pembrolizumab for the treatment of advanced biliary cancer: results from the KEYNOTE‐158 and KEYNOTE‐028 studies. Int J Cancer. 2020;147:2190‐2198. 32359091 10.1002/ijc.33013

[16] Le DT , Durham JN , Smith KN , et al. Mismatch repair deficiency predicts response of solid tumors to PD‐1 blockade. Science. 2017;357:409‐413. 28596308 10.1126/science.aan6733 PMC5576142

